# Effects of species-dominated patches on soil organic carbon and total nitrogen storage in a degraded grassland in China

**DOI:** 10.7717/peerj.6897

**Published:** 2019-05-03

**Authors:** Yujuan Zhang, Shiming Tang, Shu Xie, Kesi Liu, Jinsheng Li, Qian Chen, Ding Huang, Kun Wang

**Affiliations:** 1 Key Laboratory of Grassland Ecology and Restoration, Ministry of Agriculture, Institute of Grassland Research, Chinese Academy of Agricultural Science, Hohhot, China; 2 Department of Ecology, School of Ecology and Environment, Inner Mongolia University, Hohhot, China; 3 Department of Grassland Science, China Agricultural University, Beijing, China

**Keywords:** Monodominant species patch, Degraded grassland, Soil organic carbon, Soil total nitrogen

## Abstract

**Background:**

Patchy vegetation is a very common phenomenon due to long-term overgrazing in degraded steppe grasslands, which results in substantial uncertainty associated with soil carbon (C) and nitrogen (N) dynamics because of changes in the amount of litter accumulation and nutrition input into soil.

**Methods:**

We investigated soil C and N stocks beneath three types of monodominant species patches according to community dominance. *Stipa krylovii* patches, *Artemisia frigida* patches, and *Potentilla acaulis* patches represent better to worse vegetation conditions in a grassland in northern China.

**Results:**

The results revealed that the soil C stock (0–40 cm) changed significantly, from 84.7 to 95.7 Mg ha^−1^, and that the soil organic carbon content (0–10 cm) and microbial biomass carbon (0–10 and 10–20 cm) varied remarkably among the different monodominant species communities (*P* < 0.05). However, soil total nitrogen and microbial biomass nitrogen showed no significant differences among different plant patches in the top 0–20 cm of topsoil. The soil C stocks under the *P. acaulis* and *S. krylovii* patches were greater than that under the *A. frigida* patch. Our study implies that accurate estimates of soil C and N storage in degenerated grassland require integrated analyses of the concurrent effects of differences in plant community composition.

## Introduction

Grasslands comprise approximately 40% of the earth’s land area and play a key role in the global carbon (C) cycle (Wang & Fang, 2009). However, most grasslands have suffered C losses due to anthropogenic disturbances. Belowground C storage accounts for as much as two to four times the amount stored in living vegetation in grasslands (Post & Kwon, 2000; Percival, Parfitt & Scott, 2000). Even a minor increase in soil organic carbon (SOC) has the potential to positively affect the global atmospheric carbon dioxide concentration and the cycle (Bu et al., 2012). Therefore, it is crucial to study the changes in soil C and nitrogen (N) stocks in grassland ecosystems.

Previous studies on Inner Mongolia steppe degradation have focused on how overgrazing induces changes in plant community composition and grassland degradation (Wang & Li, 1998; Chen & Wang, 2000). Overgrazing generally results in variation in vegetation composition, a decrease in plant production, and a consequent reduction in C input (Bagchi & Ritchie, 2010). Variation in vegetation is an important factor affecting the characteristics of the soil biological community and ecosystem functions such as soil C and N storage and retention (Zeng et al., 2009; Wardle & Zackrisson, 2005). Long-term overgrazing has led to discontinuous and patchy vegetation in degraded grasslands of northern China, especially in terms of the plant dominance patterns, which historically were homogenous and dominated by *Stipa krylovii* (Zhou et al., 2007; Hirobe et al., 2013). However, it is still not clear how soil C and N stocks are distributed beneath different monodominant species in patchy degraded grassland communities.

Patchy vegetation is a very common and important phenomenon during the process of grassland degradation in typical steppes of China, and may have important consequences for C and N stocks (Erfanzadeh et al., 2014; Wang & Li, 1998; Chen & Wang, 2000). In steppe ecosystems, *Leymus chinensis* is a dominant species in the climax stage, and the grassland plant communities become dominated in the order of *S. krylovii*, *Artemisia frigida*, and *Potentilla acaulis* under continuous heavy grazing. Deterioration occurs when plant communities change from *S. krylovii* patches to *P. acaulis* patches (Wang & Li, 1998). *P. acaulis* becomes the dominant species in seriously degraded steppes (Wang & Li, 1998; Chen & Wang, 2000). However, how these patterns of variation in dominant species in plant communities influence soil C and N dynamics remains obscure.

In this study, we assessed the differences in soil C and N characteristics beneath plant community patches with different monodominant species in degraded steppe. We addressed the following two questions: (i) Do plant community patches affect soil C and N in the topsoil of degraded grassland? and (ii) Can we explain any variation in soil C and N with variations in the plant community patches?

## Materials and Methods

### Study site description

**Figure 1:**
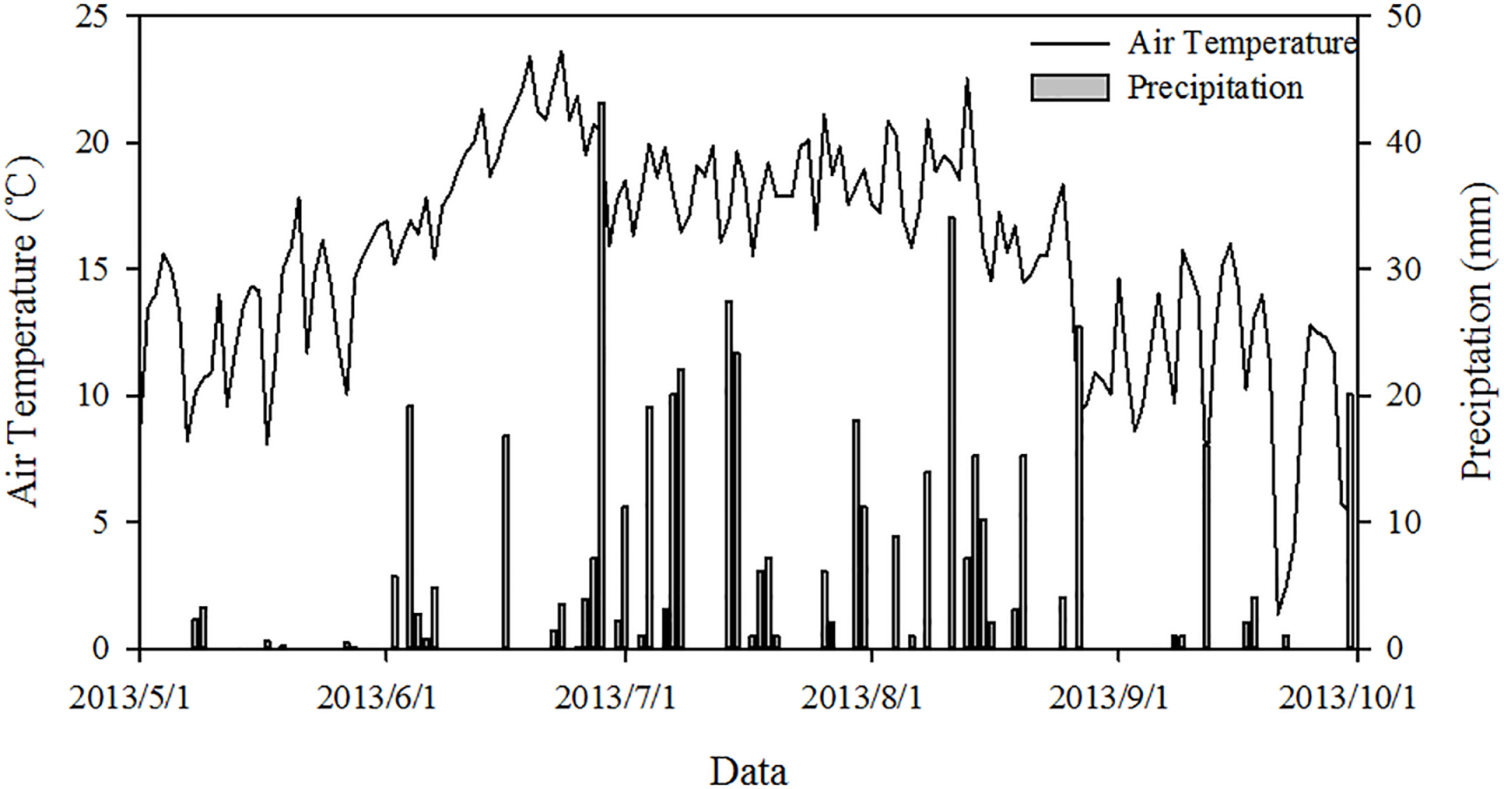
Dynamics of air temperature and precipitation in the study area from May 1 to October 1, 2013.

This field study was performed in a free-grazing area at the Field Station for Grassland Ecosystem of China Agricultural University, Guyuan County, Hebei Province, China (41°46′N, 116°16′E). The mean annual precipitation, temperature, evaporation capacity, and altitude are 430 mm, 1.4 °C, 1,700–2,300 mm, and 1,475 m, respectively. Approximately 80% of the precipitation falls during the growing season from July to September in conjunction with high temperatures. The maximum monthly mean temperature is 21.1 °C in July, and the minimum is −18.6 °C in January. The soil is chestnut soil (Chinese Soil Taxonomy) or Calciustepts soil (US Soil Taxonomy classification). The daily air temperature and rainfall distribution of the study area are shown from May 1 to October 1, 2013 (Fig. 1).

These grasslands were opened up to private use in the late 1970s, and intensive grazing resulted in plant community degradation to different extents (Yuan et al., 2005; Li et al., 2011). The total vegetation canopy was approximately 38.2%, and the plant community was primarily composed of monospecific patches of *P. acaulis*, *A. frigida*, and *S. krylovii* (Li et al., 2011). The originally dominant plants were *S. krylovii* and *L. chinensis*, which now show dwarf growth habits. In the free-grazed area, *S. krylovii*, *L. chinensis*, *Cleistogenes squarrosa* (Trin.), and other forbs were sparsely distributed and scattered inside or outside the main patches (Zhang et al., 2015; Li et al., 2011).

### Experimental investigation and sample analyses

Two key regions of degraded grasslands (with the same backgrounds of grazing history) were selected during the peak growing period for plants in August 2013. We chose three types of patches according to the community dominance in each region. Communities dominated by *S. krylovii* were called *S. krylovii* patches. Similarly, the communities dominated by *A. frigida* and *P. acaulis* were denominated as *A. frigida* patches and *P. acaulis* patches. We established one 40 × 40 m plot in the chosen degraded region and then randomly established four replicates for each monodominant species in each plot. Therefore, there were a total of 24 monospecific patches were used for this study (2 regions × 3 species patches × 4 replicates per region = 24). Each monospecific patch was 0.4–1.0 m^2^. We established one sampling plot (50 × 50 cm) in the center of each patch and evaluated the plant community structure and biomass. All plant material was clipped closely to the ground, oven-dried at 65 °C for 48 h and then weighed to determine the aboveground biomass. The belowground biomass was sampled in 20 cm increments to a depth of 40 cm using a soil auger (eight cm diameter). Three randomly located soil cores (three cm in diameter) were sampled under each quadrat in each patch to depths of 0–10, 10–20, and 20–40 cm. The three cores for a given depth within each quadrat were mixed thoroughly to obtain a composite sample. All of the soil samples were passed through a two-mm sieve to remove roots, large pieces of plant material, debris, and gravel. Then, each soil sample was divided into two parts: one part was air-dried for the analyses of soil physicochemical characteristics and the other was stored under refrigerated conditions (4 °C) for the subsequent assessment of microbial biomass carbon (MBC) and microbial biomass nitrogen (MBN).

The soil temperature (ST) and soil moisture (SM) at six cm were measured by a Delta-T soil moisture sensor (HH2; Delta-T Devices, Cambridge, UK) in August 2013. The soil pH and electrical conductivity (EC) were determined by a glass electrode (LE438, LE703 electrode, Mettler Toledo, Columbus, OH, USA) using a 1:5 soil/water suspension. The soil bulk density (BD) was determined using a coring method (Wang, Han & Li, 2008). The soil texture was measured by a laser particle analyzer (Mastersizer 2000, Malvern, England). We divided the soil particles into three parts: sand (50–200 μm), silt (2–50 μm), and clay (<2 μm). The SOC content (g kg^−1^) was determined by the H_2_SO_4_-K_2_Cr_2_O_7_ oxidation method (Nelson & Sommers, 1982). The soil total nitrogen (TN) content (g kg^−1^) was analyzed using the Kjeldahl wet digestion procedure (Gallaher, Weldon & Boswell, 1976) by a Kjeltec 2300 Analyzer (FOSS, Höganäs, Sweden). The SOC and TN stocks (Mg ha^−1^) of the 0–40 cm soil depth were calculated as follows:

$${\rm{SOC}}\;\left( {{\rm{Mg}}\;{\rm{h}}{{\rm{a}}^{ - 1}}} \right) = \sum\limits_{i = 1}^n {{\rm{B}}{{\rm{D}}_i}} \times {{\rm{D}}_i} \times {\rm{SO}}{{\rm{C}}_i} \times {10^{ - 1}}$$

$${\rm{TN}}\;\left( {{\rm{Mg}}\;{\rm{h}}{{\rm{a}}^{ - 1}}} \right) = \sum\limits_{i = 1}^n {{\rm{B}}{{\rm{D}}_i}} \times {{\rm{D}}_i} \times {\rm{T}}{{\rm{N}}_i} \times {10^{ - 1}}$$

where BD_*i*_, D_*i*_, SOC_*i*_, and TN_*i*_ represent the soil BD (g cm^−3^), depth (cm), SOC content (g kg^−1^), and TN content (g kg^−1^) of the *i*th soil layer, respectively (*i* = 1, 2, and 3).

The MBC and MBN were estimated according to the chloroform fumigation method (Brookes et al., 1985). We calculated MBC and MBN using the following formulas:

$${\rm{MBC}} = {{\left( {{C_{{\rm{fumigate}}}} - {C_{{\rm{control}}}}} \right)} \over {{K_C}}}$$

$${\rm{MBN}} = {{\left( {{N_{{\rm{fumigate}}}} - {N_{{\rm{control}}}}} \right)} \over {{K_N}}}$$

where *C*_fumigate_ and *C*_control_ represent the MBC from the fumigated samples and the control samples (unfumigated), respectively, *N*_fumigate_ and *N*_control_ represent the MBN from the fumigated samples and the control samples (unfumigated), respectively, *K*_C(N)_ is the K_2_SO_4_ extract efficiency factor for microbial C (N), and *K_C_* = 0.38 and *K_N_* = 0.54.

## Statistical Analyses

The statistical analyses were performed using SPSS 20.0 (SPSS, Chicago, IL, USA). One-way ANOVA was used to assess the differences in species richness, the SOC and TN content of the soil layers (0–10, 10–20, and 20–40 cm), the SOC and TN stocks (0–40 cm), and the MBC and MBN content (0–10 and 10–20 cm) among the three monodominant plant communities. Significant differences (*P* < 0.05) in the treatment means were determined using the least significant difference statistic for multiple comparisons. Kolmogorov–Smirnov tests were used to check for normality, and the assumption of the homogeneity of variances was checked using Levene’s test. Regression analyses were performed to evaluate the relationships between (i) the SOC and TN with sand contents and pH and (ii) SOC and TN content with MBC and MBN.

## Results

### The community characteristics of the monodominant species patches

**Figure 2:**
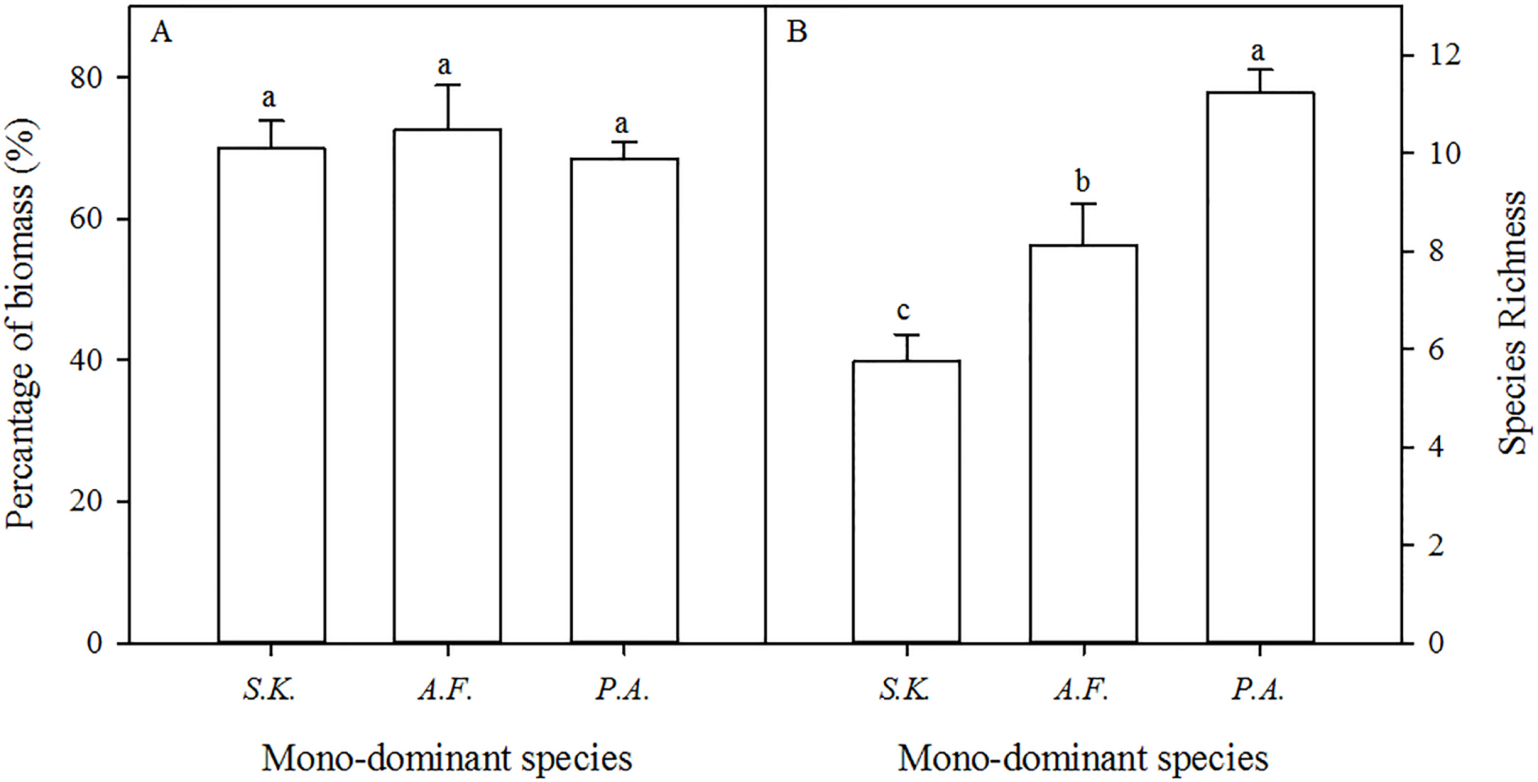
Mean percentage of total biomass represented by the dominant species (A) and the change in species richness (B) (per quadrat, means ± SD) in the mono-dominant species patches in August 2013. The different lowercase letters inside the histogram indicate significant differences among the three mono-dominant species patches (*P* < 0.05).

**Figure 3:**
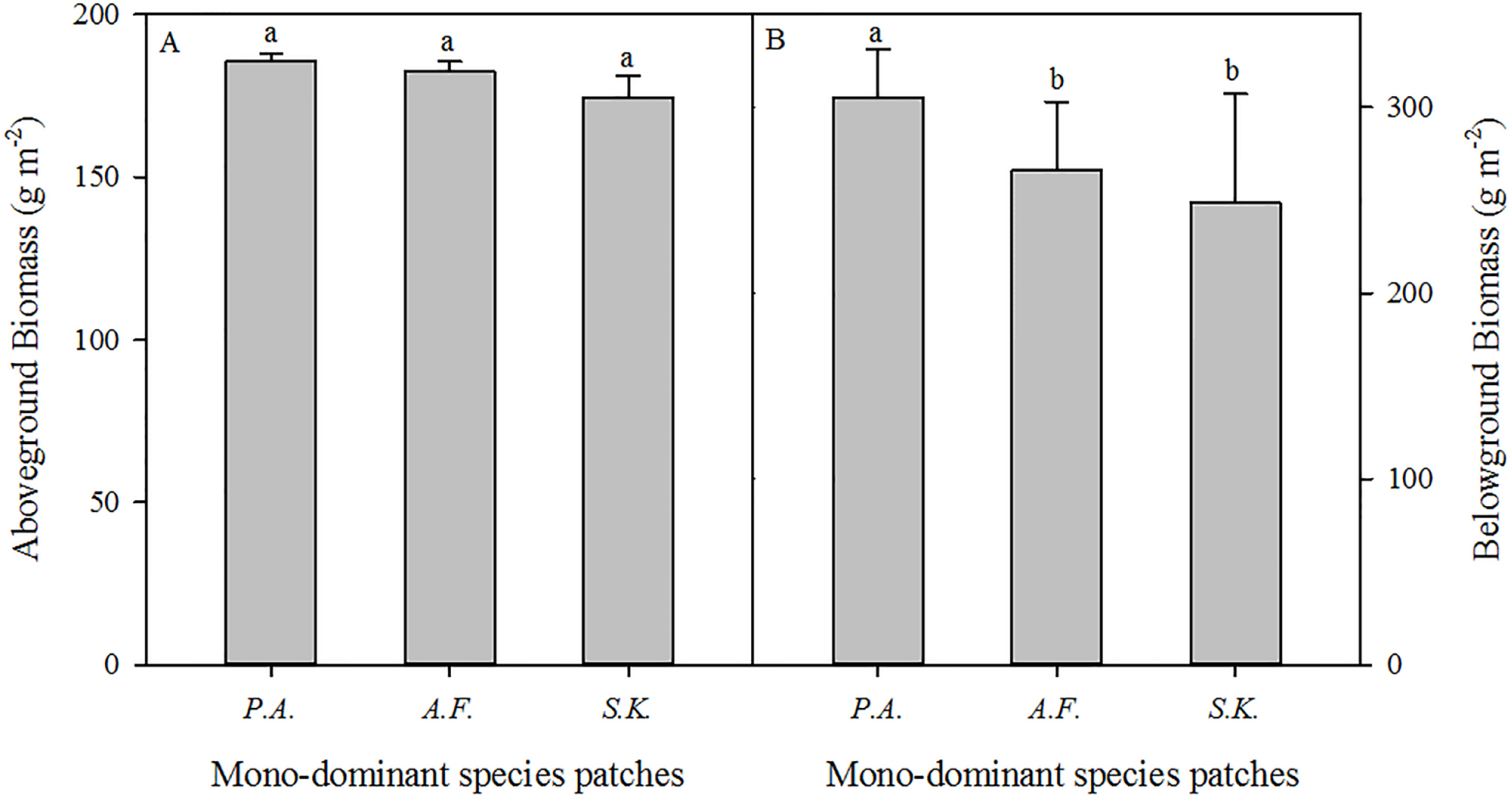
The aboveground (A) and belowground biomass (B) of three mono-dominant species patches in August 2013. The different lowercase letters inside the histogram indicate significant differences among the three mono-dominant species patches (*P* < 0.05).

In the *A. frigida*, *P. acaulis*, and *S. krylovii* patches, the percentage cover of the dominant species (*A. frigida*, *P. acaulis*, and *S. krylovii*) was 60.0%, 74.6%, and 70.1% in the sampled quadrats (Table 1). The biomass of the dominant species accounted for 72.6%, 68.5%, and 69.9% of the total biomass of the *A. frigida*, *P. acaulis*, and *S. krylovii* patches, respectively (Fig. 2A). The species richness increased significantly in the order of *S. krylovii* to *A. frigida* to *P. acaulis* (Fig. 2B). There were no significant differences in aboveground biomass among the three patch types. However, the belowground biomass was significantly higher under *P. acaulis* than under *S. krylovii* and *A. frigida*, and there was no significant difference between *S. krylovii* and *A. frigida* (Fig. 3).

**Table 1:** Percentage cover of species in three mono-dominant plant species patches.

Species	Mono-dominant species patches		
	*Stipa krylovii*	*Artemisia frigida*	*Potentilla acaulis*
*Artemisia frigida* Willd.	5.73	60.02	1.54
*Potentilla acaulis* L.	0	1.83	74.56
*Stipa krylovii* Roshev	70.11	1.26	2.24
*Leymus chinensis* (Trin.) Tzvel.	3.14	2.84	5.02
*Cleistogenes squarrosa* (Trin.) Keng	1.49	2.21	2.35
*Thalicctrum petaloideum* L.	1.01	0.46	3.05
*Sibbaldia adpressa* Bge.	0.53	0.75	0
*Saussurea amara* (L) DC.	7.45	0	0.18
*Androsace longifolia* Turcz.	1.06	3.23	2.20
*Allium tenuissimum* L.	1.12	0.67	0.89
*Astragalus tataricus* Franch.	1.44	1.45	1.84
*Cymbaria dahurica* L.	0	4.28	3.35
*Artemisia eriopoda* Bge.	3.19	2.21	1.29
*Iris lactea* Pall *var. chinensis* (Fisch).Koidz	3.85	2.66	0
*Medicago ruthenica* (L.) Trautv.	0.53	0	2.45
*Bupleurum chinense* DC.	1.06	0.48	0
*Gentiana dahurica* Fisch.	0	0.89	0
*Potentila bifurca* L. var. major Ledeb.	0	2.64	0
*Potentilla verticillaris* Steph. ex Willd.	0	0	1.69
*Polygonum sibiricum* Laxm.	0.74	0	0
Total cover	71.52	65.17	76.78
Grassland trend	Stable	Stable	Stable
Grassland condition	Degraded	Degraded	Degraded

### Soil physical characteristics

Although the three types of patches were in close proximity to each other, the soil physical characteristics varied with the dominant plant species. The SM (zero to six cm) under *P. acaulis* was greater than that under the other species. The ST (zero to six cm) was 25.5 °C under *P. acaulis*, which was 0.4 and 0.6 °C less than under *A. frigida* and *S. krylovii*, respectively. The soil pH, EC, and BD differed among the patches and soil depths, generally increasing with increasing soil depth (Table 2).

**Table 2:** Soil physical characteristics of three different dominant species patches.

Patches	0–6 cm		0–10 cm			10–20 cm			20–40 cm		
	SM (%)	ST (°C)	PH	EC (ms m^−1^)	BD (g cm^−3^)	PH	EC (ms m^−1^)	BD (g cm^−3^)	PH	EC (ms m^−1^)	BD (g cm^−3^)
*Potentilla acaulis*	12.60	25.45	8.03	179.00	1.17	8.56	172.80	1.62	9.62	387.40	1.70
*Artemisia frigida*	11.23	26.07	8.23	152.80	1.20	8.40	188.20	1.70	9.64	454.20	1.51
*Stipa krylovii*	11.31	25.92	8.37	138.30	1.26	8.95	475.60	1.82	9.93	802.80	1.77

**Note:**

The data shown in Table 3 were mean. SM, soil moisture; ST, soil temperature; EC, electrical conductivity; BD, bulk density.

The soil texture was also affected by the dominant plant species and soil depth (Table 3). In the upper layer (0–10 cm), for instance, the sand content beneath the *A. frigida* patches was greater than that under the *P. acaulis* and *S. krylovii* patches (58.6%, 54.0%, and 56.1%, respectively; *P* < 0.05). The silt and clay contents were generally greater under *P. acaulis* compared with the contents under the other species. There were no differences between the silt content under the *P. acaulis* and *S. krylovii* patches or between the clay content under the *A. frigida* and *S. krylovii* patches at the 0–10 cm soil depth.

**Table 3:** Percentage of sand, silt, and clay of soil beneath the three different mono-dominant species patches.

Layer (cm)	Soil texture	*Potentilla acaulis*	*Artemisia frigida*	*Stipa krylovii*
0–10	Sand	54.00 ± 0.86^b^	58.61 ± 0.42^a^	56.12 ± 0.78^b^
	Silt	42.54 ± 0.86^a^	38.30 ± 0.39^b^	40.73 ± 0.76^a^
	Clay	3.70 ± 0.07^a^	3.08 ± 0.10^b^	3.15 ± 0.07^b^
10–20	Sand	61.69 ± 1.08^a^	63.35 ± 1.41^a^	60.59 ± 0.80^a^
	Silt	35.93 ± 1.16^a^	34.23 ± 1.24^a^	37.02 ± 0.82^a^
	Clay	2.37 ± 0.18^a^	2.42 ± 0.18^a^	2.39 ± 0.14^a^
20–40	Sand	62.80 ± 1.32^a^	59.91 ± 0.83^ab^	57.33 ± 1.51^b^
	Silt	32.13 ± 0.94^b^	38.00 ± 0.91^a^	39.39 ± 1.53^a^
	Clay	5.07 ± 0.39^a^	2.09 ± 0.17^c^	3.27 ± 0.26^b^

**Note:**

The data are presented as mean ± SE. Different lowercase letters in the same row indicate significant differences at 0.05 level.

### SOC, TN, MBC, and MBN beneath the patches

**Figure 4:**
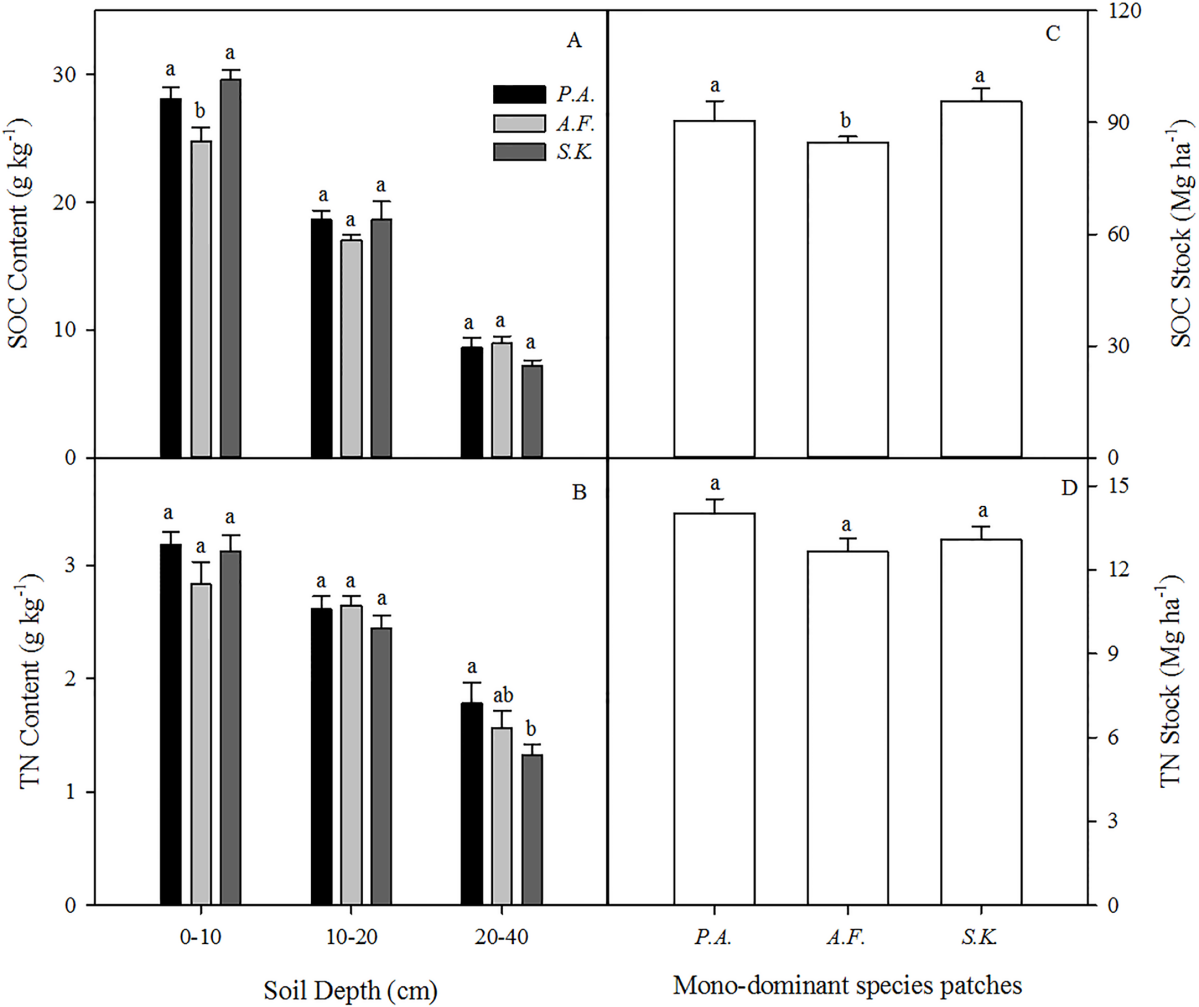
Mean soil organic carbon (SOC) (A) and total nitrogen (TN) (B) contents (g kg^−1^) in the 0 –10, 10–20, and 20–40 cm soil layers and the SOC stock (Mg ha^−1^) (C) and TN stock (Mg ha^−1^) at 0–40 cm (D) under three different plant species in 2013. Data are shown as the mean ± SE. Lower case letters indicate differences among species at the same soil depth (*P* < 0.05). *P.A., Potentilla acaulis* patch; *A.F., Artemisia frigida* patch; *S.K., Stipa krylovii* patch.

The SOC content and stocks under *A. frigida* were significantly lower than those under *P. acaulis* and *S. krylovii* However, there was no effect of species on SOC content or stocks in the deeper soil layers (10–20 and 20–40 cm) (Figs. 4A and 4C). The soil TN content decreased with soil depth, but the dominant plant species did not affect the TN content except in the 20–40 cm soil layer (Fig. 4B). Moreover, the dominant plant species patches had no effect on the soil TN stock (Fig. 4D).

**Figure 5:**
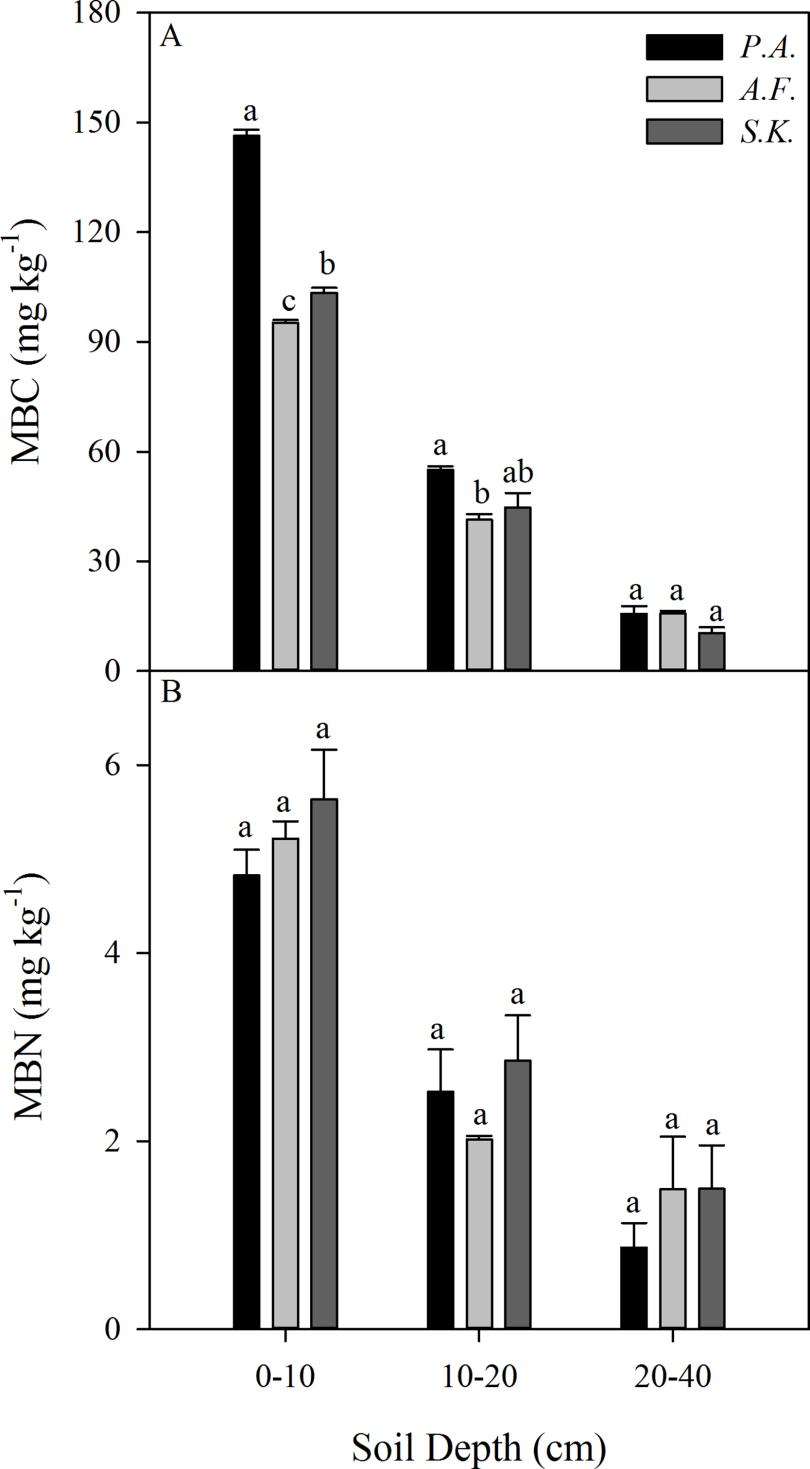
Mean microbial biomasscarbon (MBC) (A) and microbial biomass nitrogen (MBN) (B) content (g kg^–1^) in the 0–10, 10–20, and 20–40 cm soil layers under three different plant species in 2013. Data are shown as the mean ± SE. Lower case letters indicate differences among species at the same soil depth (*P* < 0.05). *P.A., Potentilla acaulis* patch; *A.F., Artemisia frigida* patch; *S.K., Stipa krylovii* patch.

The MBC content (mg kg^−1^) was influenced by the dominant plant species in the 0–10 and 10–20 cm soil layers, but not in the 20–40 cm soil layer. However, there was no difference among the species in terms of the MBN content (mg kg^−1^) in any soil layer from 0–40 cm (Fig. 5).

### Correlations of SOC and TN with other factors

**Figure 6:**
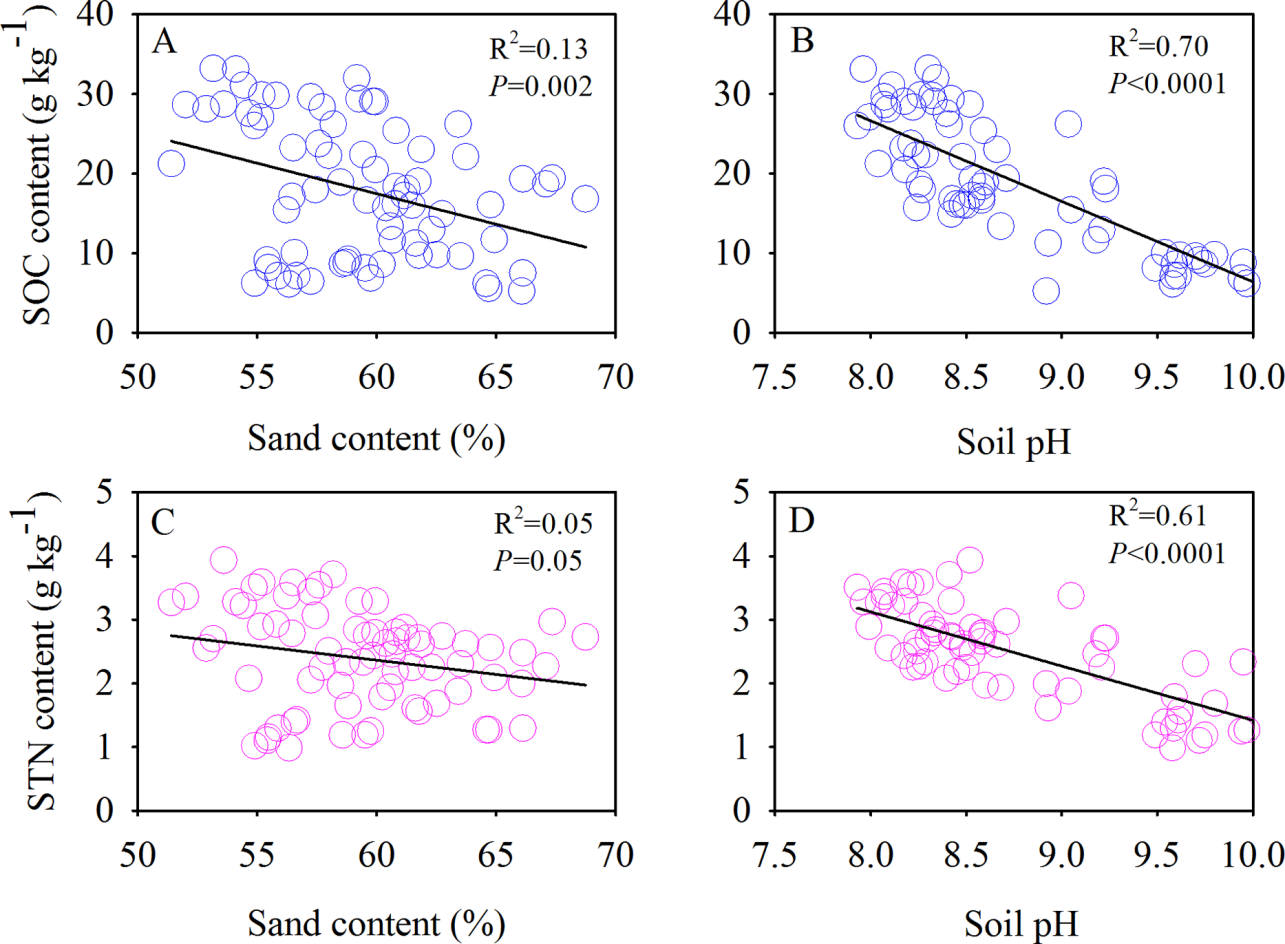
Correlations of SOC content with sand content (A) and soil pH (B), and STN content with sand content (C) and soil pH (D), respectively.

**Figure 7:**
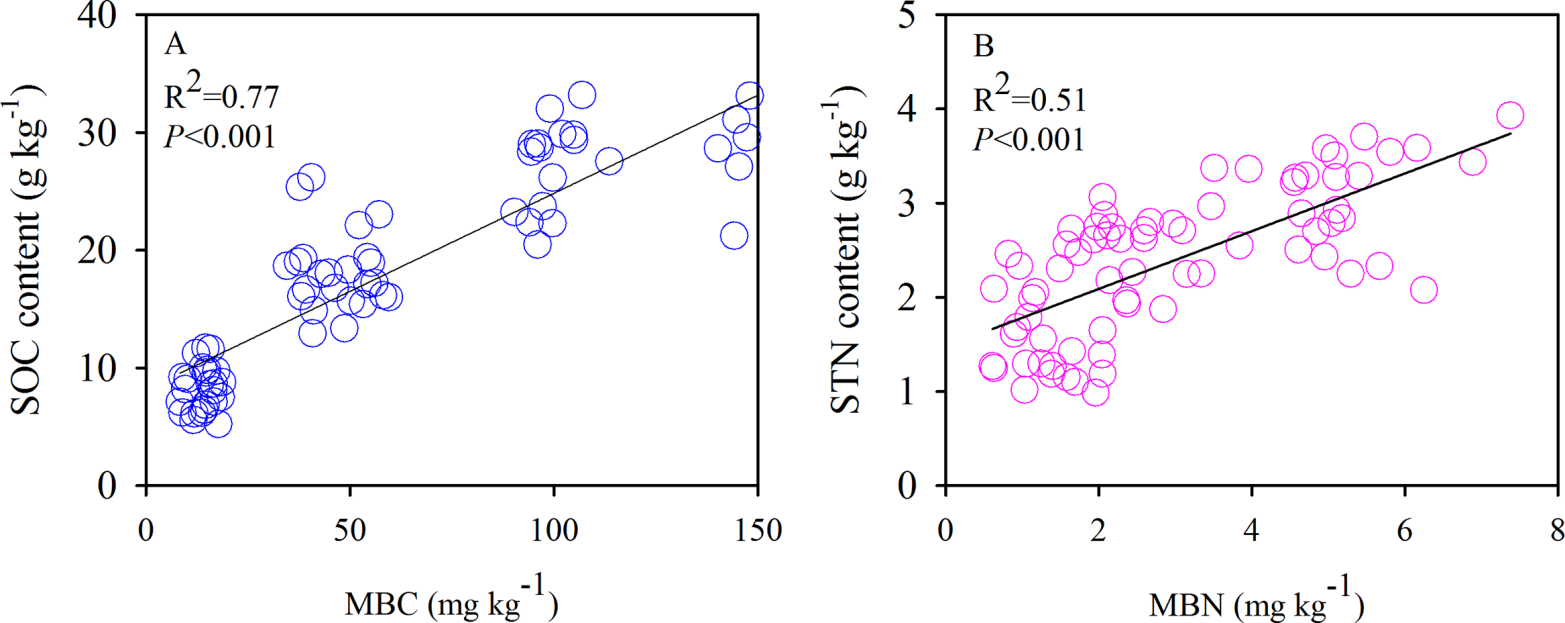
Correlations of SOC content with MBC (A), and STN content with MBN (B), respectively.

The SOC and TN contents showed significant negative correlations with the soil sand content and pH (Fig. 6). Moreover, we also found significant positive correlations between the SOC and MBC (Fig. 7A) and similar correlations between the soil TN and MBN under different species patches (Fig. 7B).

## Discussion

Aboveground plant community patches can potentially influence the biotic and abiotic properties of soil in natural ecosystems (Solon, Degórski & Roo-Zielińska, 2007). In this study, we observed that soil physical and chemical properties differed beneath the three monodominant species patches (Tables 2 and 3; Figs. 4 and 5). These results are generally consistent with those found in other studies (Solon, Degórski & Roo-Zielińska, 2007; Post & Kwon, 2000). Increasing the C input and retention by plants and reducing the SOC turnover can enhance soil C sequestration. Moreover, in arid and semiarid grassland ecosystems, soil nutrients may accumulate as fertile islands under vegetation patches (Reynolds et al., 1999; Jackson et al., 2002). The variation in vegetation types may be the main driving factor regulating C and N dynamics and the spatial heterogeneity of soil nutrient content (Schlesinger et al., 1996; Jiang et al., 2011). Jiang et al. (2011) found that plant species could account for 72% of the variation in the SOC concentration and 77% of the variation in the TN concentration. In this study, the species richness of the *P. acaulis* patches was significantly higher than that of the *A. frigida* and *S. krylovii* patches. The *P. acaulis* community generated more primary production, and its belowground biomass was significantly higher than that of the *A. frigida* and *S. krylovii* communities. This indicates that the dominant plant species regulated the quantity and quality of the litter returned back into the soil to impact the soil C and N properties (Eviner & Chapin, 2006). However, vegetation-soil feedback mechanisms are complex; depend on the plant species, functional groups, and site-specific differences; and regulate vegetation community development and soil formation (Puigdefabregas et al., 1999; Wu et al., 2011). Therefore, further studies are needed in the future on vegetation-soil feedback mechanisms under various plant patches.

Individual plant species differ in their aboveground and root biomass, litter quality, root exudation, efficiency of nutrient acquisition (Eviner & Chapin, 2006), and associated microbial diversity and activity (Garbeva, Van Elsas & Van Veen, 2008). In our study, differences in the structure of the monodominant species community showed strong impacts on SOC, TN, and MBC. Our results showed that the SOC content of the 0–10 cm layer, the TN content of the 20–40 cm layer, the SOC stock of the 0–40 cm layer, and the MBC content of the 0–10 and 10–20 cm layers diverged among the species patches (Figs. 4 and 5). However, the reason that there were no differences in MBN or TN (0–10 and 10–20 cm soil layers and TN stock) among the three plant communities needs further investigation. In general, greater C and N accumulation were measured for *P. acaulis* and *S. krylovii* than for *A. frigida* Plant-specific influences on soil processes may be explained by plant-specific effects on soil microhabitat conditions and the quality and quantity of primary production that plants return to the soil (Viketoft et al., 2009). In semiarid areas, erosion is the predominant factor causing SOC loss (Martínez-Mena et al., 2002). The current study showed that the amount of soil particles was different under various plant patches but was which closely correlated with SOC and TN content (Table 2; Fig. 6). Generally, soil with a fine texture stores more C than coarse soil because small particles and lower porosity restrict soil microbial activity and protect nonlabile organic matter from decomposition (Oades, 1988). Moreover, we found that changes in soil pH could affect SOC and TN contents after grassland degradation, which would impact the soil C and N process and the microbial activities that are responsible for SOC and TN stocks. Furthermore, changes in soil microbes could affect SOC and TN losses under different plant patches. This may be due to increases in soil compaction and BD by long-term livestock trampling, which causes microorganisms to use less of the available carbon energy and reduces soil organic matter storage in degraded steppe landscapes (Wu et al., 2010). Our results also showed that the plant patch-induced changes in MBC and MBN were positively linearly correlated with those in SOC and TN.

In addition, the morphological characteristics of plants may be a main reason for the variation in soil C and N. Previous research has shown that *P. acaulis* takes advantage of a broad, waist-inverted centrum three-dimensional root architecture to hold rainwater and improve water use efficiency and then increases its aboveground biomass to sequester more C from the atmosphere under extremely arid conditions (Zhou & Wang, 2011). Additionally, *P. acaulis* belongs to a group of perennial and stemless xerophytes with clonal reproduction. The plant leaves closely cover the soil surface, preventing wind erosion while at the same time decreasing soil water evaporation. These responses may also be related to the low level of animal preference for *P. acaulis*, which affects the biogeochemistry of the ecosystem and results in greater carbon and nutrient accumulation in aboveground biomass and more litter return to soil than would be expected, thereby having a positive effect on the SOC content. This was consistent with previous research (Guo, Wang & Gifford, 2007; Wu et al., 2008) in which it was shown that the grazing avoidance of *P. acaulis* minimized livestock disturbance. The reduction in livestock trampling and foraging in *P. acaulis* patches along with the morphological characteristics of its root system may be the main reason why there was greater soil C accumulation under *P. acaulis* patches. In fact, *P. acaulis* is seen as an indicator species of the last successional stage in degraded grasslands, but it was shown to play an important ecological function by increasing SOC accumulation and improving the sustainability of the soil system in this study.

## Conclusions

Plant community patches can potentially influence the biotic and abiotic properties of soil in natural ecosystems. The results showed that soil C and N patterns had different responses to three monodominant species patches in degraded grassland. Specifically, the soil C stock, SOC and MBC contents varied significantly among the three monodominant species patches. Ecologically, *P. acaulis*, as a plant indicator of severely degraded grassland, served an important ecological function by taking advantage of its special root architecture, clonal reproduction characteristics and low level of animal preference to improve soil texture and soil C stocks to a greater degree than the other two species.

## Supplemental Information

10.7717/peerj.6897/supp-1 Supplemental Information 1 Raw data. The raw data for vegetation community characteristics and soil physicochemical of three specie-dominated patches.

## Additional Information and Declarations

### Data Availability

The following information was supplied regarding data availability: Raw data are available as a Supplemental File.
